# Effects of Celery, Rosehip, and Blackcurrant Powders on Quality Attributes of Salted and Unsalted Sous-Vide Pheasant Meat During Refrigerated Storage

**DOI:** 10.3390/foods15132369

**Published:** 2026-07-03

**Authors:** Georgiana Ancuța Mîșu, Raluca-Ștefania Rădoi-Encea, Roxana-Andreea Munteanu-Ichim, Ana-Maria Radu, Alina Maier, Cristina Maria Canja, Mirabela Ioana Lupu, Florentina Matei

**Affiliations:** 1 Faculty of Biotechnology, University of Agronomic Sciences and Veterinary Medicine of Bucharest, 59 Mărăști Blvd., District 1, 011464 Bucharest, Romania; misu.anca@yahoo.com (G.A.M.); roxana.ichim@unitbv.ro (R.-A.M.-I.); anamaria.manolica@unitbv.ro (A.-M.R.); florentina.matei@unitbv.ro (F.M.); 2 Faculty of Food and Tourism, Transilvania University of Brașov, 148 Castelului St., 500014 Brașov, Romania; alina.maier@unitbv.ro (A.M.); canja.c@unitbv.ro (C.M.C.); lupu.mirabela@unitbv.ro (M.I.L.)

**Keywords:** sous-vide, pheasant meat, plant powder, clean-label meat, refrigerated storage, lipid oxidation

## Abstract

Celery (*Apium graveolens*), rosehip (*Rosa canina*), and blackcurrant (*Ribes nigrum*) powders (1–3%, *w*/*w*) were evaluated in sous-vide pheasant meat prepared with or without 1.8% NaCl and stored for 28 days at 4 °C. Measurements included proximate composition, pH and titratable acidity, instrumental texture and color, FRAP, ABTS, Folin–Ciocâlteu-reactive compounds, TBARS, nitrate/nitrite, chemical-spoilage screening, and microbial counts; sensory quality was assessed descriptively by a laboratory panel on day 28. Mean cooking loss was 22.11% in salted and 24.10% in unsalted samples. At day 28, 3% rosehip and blackcurrant formulations had total viable counts of 3.80–4.16 log_10_ CFU/g, compared with 5.78–6.28 log_10_ CFU/g in controls. Their FRAP values were 2.83–3.23 µmol TE/g dry matter and TBARS values were 0.43–0.57 mg MDA/kg, compared with 1.00–1.05 µmol TE/g dry matter and 1.45–1.53 mg MDA/kg in controls. Celery at 3% retained approximately 170 mg/kg nitrate and 13.3–13.4 mg/kg nitrite. Rosehip and blackcurrant, particularly at 3%, provided the most favorable combination of measured quality attributes under the tested conditions. These findings demonstrate ingredient-specific effects but do not validate pathogen control, commercial shelf life, or population-level consumer preference.

## 1. Introduction

The common pheasant (*Phasianus colchicus*) provides a lean meat characterized by high protein and low fat contents. Reported values for breast meat commonly fall within approximately 20–27 g protein/100 g, 0.8–1.3 g fat/100 g, and 71–73 g water/100 g [1,2,3]. Its limited intramuscular fat and the structure of game muscle may nevertheless restrict juiciness and tenderness, making processing conditions particularly important for product quality [4]. Sous-vide processing combines vacuum packaging with accurately controlled heating and may reduce moisture loss and improve texture relative to conventional cooking, especially in lean meat matrices [5,6,7].

The quality of refrigerated sous-vide products remains dependent on formulation, thermal history, storage temperature, and storage duration [8,9,10]. Plant-derived ingredients have therefore received attention as components of clean-label, multi-hurdle preservation strategies. Celery powder is used as a vegetable-derived nitrate source in naturally cured meat products [11,12]. Rosehip and blackcurrant powders are reported to contain organic acids and phenolic compounds and have been evaluated as quality-modulating ingredients in meat systems [13,14,15,16]. Fruit-derived powders, pomaces, and extracts may also influence water retention, texture, color, reducing capacity, lipid oxidation, and microbial development, although their effects depend strongly on the plant matrix, dose, meat species, and processing conditions [17,18,19,20].

Comparative evidence remains limited for a lean game-meat matrix processed by sous-vide, particularly when a nitrate-oriented vegetable powder and acid- and phenolic-rich fruit powders are evaluated at several inclusion levels under both salted and unsalted conditions. The present study therefore compared celery, rosehip, and blackcurrant powders at 1%, 2%, and 3% in sous-vide pheasant meat during 28 days of refrigerated storage. The inclusion range was selected to examine dose-dependent responses within a technologically manageable range while limiting excessive displacement of the meat matrix. NaCl was included at 1.8% to represent a moderate level relevant to cooked meat products and to test whether ionic-strength and water-binding effects modified the response to the plant powders.

The objectives were to determine the effects of plant-powder type, inclusion level, NaCl condition, and storage time on physicochemical composition, texture, instrumental color, reducing and radical-scavenging capacities, lipid oxidation, nitrate/nitrite behavior, chemical-spoilage indicators, microbial development, and laboratory-panel sensory scores. It was hypothesized that celery would primarily increase nitrate/nitrite availability, whereas rosehip and blackcurrant would exert broader effects on acid–base status, antioxidant-related measurements, lipid oxidation, and refrigerated quality.

## 2. Materials and Methods

### 2.1. Raw Materials, Reagents, and Powder Characterization

Pheasant meat portions were obtained from legally managed Romanian hunting grounds associated with Transilvania University of Brașov, transported under refrigeration, and stored at 4 °C until processing. Commercial food-grade celery (*Apium graveolens*), rosehip (*Rosa canina*), and blackcurrant (*Ribes nigrum*) powders were purchased in Brașov, Romania and kept in sealed, light-protected containers. The powders were characterized before formulation using the analytical procedures described below. Their measured composition is summarized in Table 1. The use of commercial powders improves practical relevance but also makes the findings specific to the investigated product batches.

### 2.2. Experimental Design and Sample Preparation

Two formulation series were prepared: a salted series (S) containing 1.8 g NaCl/100 g raw meat and an unsalted series (NS) without added NaCl. Each series comprised a control and celery, rosehip, or blackcurrant powder at 1%, 2%, or 3% (Table 2). Each formulation was prepared from 100 g portions of raw pheasant meat. For every formulation and storage time, three separately prepared and vacuum-packaged portions constituted the experimental units (*n* = 3). Replicate analytical readings obtained from the same package were averaged before statistical analysis and were not treated as independent observations. The experiment was conducted in one production campaign. ABTS, TBARS, powder characterization, and instrumental color were measured during that campaign using the original experimental materials and sample units. The single-campaign design is acknowledged as a limitation for broader batch-to-batch inference.

Sample preparation was performed under hygienic conditions in a laminar-flow hood. Uniform meat portions were combined with the designated plant powder and, where applicable, NaCl; transferred to multilayer PA/PE sous-vide bags (75 µm total thickness; HENDI, De Klomp, The Netherlands); vacuum packaged; and heated at 65 °C for 90 min in a water bath equipped with an iVide Plus circulator (HENDI). The bags were cooled in ice water to below 10 °C within 30 min and stored at 4 °C. Independent packages were randomly allocated to days 0, 7, 14, 21, and 28, and refrigerator positions were rotated periodically.

### 2.3. Physicochemical and Proximate Analyses

#### 2.3.1. pH and Titratable Acidity

A 10 g portion of the drained cooked sample was homogenized with 90 mL distilled water for 60 s. The pH of the aqueous homogenate was measured at 20 ± 2 °C according to ISO 2917:1999 [21] using a calibrated digital pH meter equipped with a glass electrode (Hanna Instruments, Cluj, Romania). Calibration was performed before each series with pH 4.00, 7.00, and 10.00 buffers. Titratable acidity was determined on the same type of homogenate by titration with standardized 0.1 N NaOH to pH 8.3 and expressed as a percentage of lactic acid equivalent [22].

#### 2.3.2. Moisture and Cooking Loss

Moisture was determined by drying to constant mass according to ISO 1442:2023 [23]. Cooking loss was calculated from the net masses of the sample before cooking and the cooked product after draining the pouch exudate [24].

#### 2.3.3. Ash, Protein, and Fat

Ash was determined by incineration in a muffle furnace according to ISO 936:1998 [25] and calculated as ash mass divided by initial sample mass. Protein was determined by the Kjeldahl procedure according to ISO 937:2023 [26]. Approximately 1 g sample was digested with 15 mL sulfuric acid (95–98%) and a potassium sulfate/copper sulfate catalyst mixture, alkalized with 40% (*w*/*v*) NaOH, distilled into 4% (*w*/*v*) boric acid, and titrated with standardized 0.1 M HCl. Crude protein was calculated as total nitrogen × 6.25. Total fat was determined by petroleum-ether extraction according to ISO 1443:1973 [27] and calculated as extracted fat mass divided by initial sample mass. All proximate results were expressed on a wet-mass basis.

### 2.4. Reducing Capacity, Radical-Scavenging Capacity, Phenolic-Reactive Compounds, and Lipid Oxidation

#### 2.4.1. Sample Extraction

For FRAP, ABTS, and Folin–Ciocâlteu analyses, homogenized sample was extracted with 80% methanol, centrifuged, and the supernatant analyzed. Matrix-specific blanks were included for colored extracts. Results were corrected using the corresponding blank response.

#### 2.4.2. FRAP and ABTS Assays

Ferric-reducing capacity was determined using freshly prepared FRAP reagent containing acetate buffer (300 mM, pH 3.6), 10 mM TPTZ in 40 mM HCl, and 20 mM FeCl_3_·6H_2_O at a 10:1:1 ratio, following Benzie and Strain [28] and Mouhoubi et al. [29]. After reaction for 10 min at 37 °C, absorbance was recorded at 593 nm using a Synergy HTX multimode microplate reader (Agilent Technologies, Santa Clara, CA, USA). ABTS radical-cation scavenging capacity was measured following Re et al. [30]. The ABTS radical cation was generated from 7 mM ABTS and 2.45 mM potassium persulfate for 12–16 h in darkness, diluted to an absorbance of 0.70 ± 0.02 at 734 nm, and reacted with sample extract for 6 min before absorbance was read on the same instrument. Trolox was the calibration standard for both assays, and results were expressed as µmol TE/g dry matter.

#### 2.4.3. Folin–Ciocâlteu-Reactive Compounds

Folin–Ciocâlteu-reactive compounds were measured according to Singleton and Rossi [31]. A 0.5 mL aliquot of extract was mixed with diluted Folin–Ciocâlteu reagent (1:10) and 7.5% Na_2_CO_3_. After 30 min in darkness, absorbance was measured at 765 nm using the Synergy HTX multimode microplate reader. Results were expressed as mg gallic acid equivalents (GAE)/100 g sample. Because the Folin–Ciocâlteu reagent is not specific exclusively to phenolics, the results are referred to as phenolic-reactive compounds rather than direct quantification of individual phenols.

#### 2.4.4. Thiobarbituric Acid-Reactive Substances

Lipid oxidation was assessed by the TBARS procedure adapted from Pikul et al. [32]. A 5 g sample was homogenized with 25 mL 7.5% trichloroacetic acid, filtered, combined with 0.02 M thiobarbituric acid, heated at 95 °C for 30 min, cooled, and measured at 532 nm using the Synergy HTX multimode microplate reader. 1,1,3,3-Tetraethoxypropane was used for calibration, and results were expressed as a mg malondialdehyde (MDA)/kg sample.

### 2.5. Nitrate, Nitrite, Sodium Chloride, and Chemical-Spoilage Evaluation

#### 2.5.1. Nitrite and Nitrate

Nitrite and nitrate were determined according to ISO 2918:1975 [33] and ISO 3091:1975 [34], respectively. Both reference methods were confirmed by ISO in 2024 and therefore remain current, although newer ion-chromatographic alternatives are available. Ten grams of homogenized sample were extracted with hot distilled water (80–90 °C; 1:10, *w*/*v*), clarified with Carrez I and II solutions, and filtered. Nitrite was quantified after diazotization with sulfanilamide and N-(1-naphthyl)ethylenediamine dihydrochloride at 538 nm. Nitrate was reduced to nitrite using a cadmium reduction column and quantified by the same chromogenic reaction. Results were expressed as mg/kg sample.

#### 2.5.2. Sodium Chloride

Sodium chloride was determined in the drained cooked tissue by aqueous extraction and gravimetric precipitation of chloride as AgCl [35]. The result was calculated stoichiometrically from the AgCl mass and expressed as NaCl percentage. Because the analytical sample excluded the pouch exudate, analytically recovered NaCl in cooked tissue was expected to be lower than the nominal 1.8% added to the raw formulation.

#### 2.5.3. Chemical-Spoilage Evaluation

Ammonia-related deterioration was screened using Nessler reagent following Zhang et al. [36]. Equal volumes of clarified sample extract and reagent were combined, allowed to react for precisely 10 min at room temperature, and evaluated on an ordinal scale: 0, negative; 1, weak; 2, moderate; and 3, strong reaction. The endpoint was interpreted descriptively and was not treated as a quantitative oxidation marker.

### 2.6. Texture Analysis

Warner–Bratzler shear force was measured using a texture analyzer equipped with a Warner–Bratzler blade (Zwick/Roell, Ulm, Germany), following Kurp et al. [37]. Standardized sample strips were sheared perpendicular to the muscle fibers, and maximum force was recorded in newtons.

### 2.7. Color Evaluation

Surface color was measured using a calibrated portable tristimulus colorimeter under D65 illumination, a 10° standard observer, and an 8 mm measurement aperture. CIE L* (lightness), a* (redness), and b* (yellowness) coordinates were recorded according to ISO/CIE 11664-4:2019 [38]. Three readings were obtained at distinct positions on each independent sample and averaged before analysis.

### 2.8. Microbiological Analysis

Microbiological analyses were conducted on days 0, 7, 14, 21, and 28. A 25 g sample was homogenized with 225 mL buffered peptone water, and decimal dilutions were prepared according to ISO 6887-2:2017 [39]. Total viable count (TVC) was enumerated on Plate Count Agar by the pour-plate technique at 30 °C for 72 h according to ISO 4833-1:2013/Amd.1:2022 [40]. Yeasts and molds were enumerated on Potato Dextrose Agar after exactly 5 days at 25 °C. Total coliform bacteria were enumerated by surface plating 0.1 mL on MacConkey agar at 37 °C for 24 h [41]. *Clostridium perfringens* was enumerated by pour plating 1 mL in TSC agar under anaerobic conditions at 37 °C for 20 h according to ISO 15213-2:2023 [42], and coagulase-positive staphylococci were enumerated by surface plating 0.1 mL on Baird–Parker agar according to EN ISO 6888-1:2021 [43]. Quantifiable TVC and yeast/mold results were expressed as log_10_ CFU/g. For solid samples yielding no colonies at the lowest dilution, method-specific reporting limits corresponded to <2 log_10_ CFU/g for total coliform bacteria and coagulase-positive staphylococci (0.1 mL surface-plating methods) and <1 log_10_ CFU/g for *C. perfringens* (1 mL pour-plating method). Results below these limits were treated as censored observations and were not entered into parametric comparisons.

*Salmonella* spp. were tested by ISO 6579-1:2017 [44], and *Listeria monocytogenes*/*Listeria* spp. were tested by the detection procedure in ISO 11290-1:2017 [45]. Detection results were reported per 25 g. The microbiological study was designed as refrigerated-quality screening and not as a pathogen challenge test.

### 2.9. Exploratory Laboratory-Panel Sensory Evaluation

An exploratory laboratory-panel assessment was conducted on day 28, the terminal point of the predefined refrigerated-storage period, to compare formulations after the longest interval tested. The component was designed as terminal formulation screening rather than longitudinal monitoring of sensory change. Assessing all 20 formulations at each of the five storage times would have required 100 product presentations per assessor and would have increased panel fatigue, adaptation, and carry-over. Ten adult graduate students enrolled in a sensory-evaluation course served as the laboratory panel. The same assessors evaluated the unsalted and salted series in separate sessions to reduce carry-over associated with salt perception, and they completed two orientation sessions to standardize the interpretation of tenderness, juiciness, flavor, and overall sensory quality. Samples were coded and randomized, reheated in sealed bags at 50 °C for approximately 10 min, and served in individual booths. Water and unsalted crackers were provided between samples. A 9-point structured quality scale was used, where 1 represented very poor quality and 9 represented excellent quality, in accordance with the principles of ISO 4121:2003 [46].

The samples were prepared and stored under the same controlled protocol as the corresponding analytical samples. Parallel microbiological analyses conducted on day 28 did not detect the targeted pathogens under the analytical conditions used; however, these analyses were not a pathogen-challenge study and do not validate product safety or a 28-day commercial shelf life. The sensory assessment was exploratory and was not designed as a population-level consumer study. Written informed consent was obtained from all participants. Sensory records were coded without direct identifiers, and consent documentation was stored separately from the analytical dataset.

### 2.10. Statistical Analysis

Results are expressed as mean ± standard deviation. For storage-dependent endpoints, a balanced three-factor ANOVA was performed with NaCl condition, formulation, storage day, and all interactions as fixed effects. The independently packaged portion was the experimental unit (*n* = 3 per NaCl condition × formulation × day). Cooking loss was analyzed by two-factor ANOVA. Where appropriate, Tukey’s honestly significant difference test was used for pairwise comparisons at *p* < 0.05. Nessler scores were treated as ordinal and summarized descriptively. Quantifiable TVC and yeast/mold counts were analyzed after log_10_ transformation. Method-censored total coliform, *C. perfringens*, and coagulase-positive staphylococcal results were summarized descriptively and were not entered into parametric comparisons. Package-level observations, not replicate analytical readings, entered the inferential models.

Laboratory-panel results are reported as means ± standard deviations for the ten assessors within each session. Because the panel was small, all assessors evaluated every formulation, and the assessment was intended as exploratory terminal screening, sensory data were interpreted descriptively. No confirmatory within-session or between-session sensory inference was made, and no conclusion was drawn regarding a NaCl main effect, a NaCl × formulation interaction, or population-level consumer preference. The remaining analyses were conducted in Python3.11 using SciPy and statsmodels.

## 3. Results and Discussion

### 3.1. Interrelationship Between pH and Titratable Acidity

**Figure 1:**
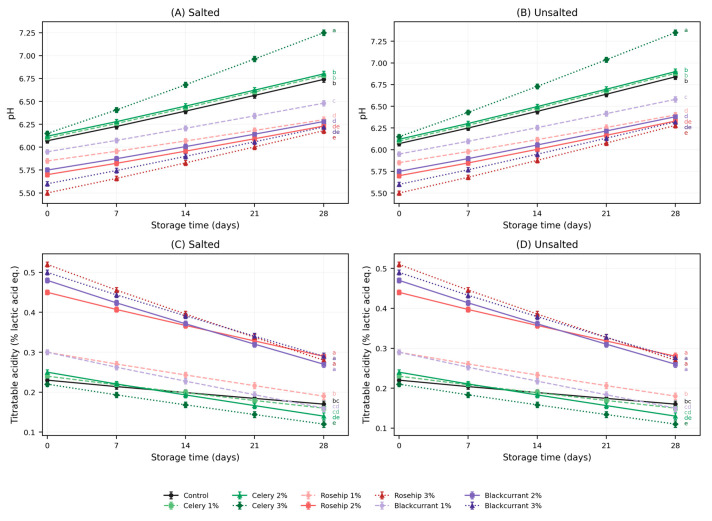
Changes in pH (**A**,**B**) and titratable acidity (**C**,**D**) of salted and unsalted sous-vide pheasant meat during refrigerated storage. Values are means ± SD of three independently packaged samples. Letters adjacent to the day-28 values indicate Tukey groupings within the corresponding NaCl series (*p* < 0.05).

pH and titratable acidity changed in opposite directions during storage (Figure 1). NaCl condition, formulation, and storage day significantly affected pH and titratable acidity (all *p* < 0.001). For pH, the NaCl × day and formulation × day interactions were significant (*p* < 0.001), whereas NaCl × formulation was not. For titratable acidity, formulation × day was significant (*p* < 0.001), but NaCl × day and NaCl × formulation were not significant. The interaction pattern further indicates that pH and titratable acidity captured related but non-interchangeable aspects of acid–base change.

pH reflects hydrogen-ion activity, whereas titratable acidity reflects the total acid reserve and buffering capacity of the protein-rich matrix. Consequently, the two parameters are generally inversely associated but need not change in a perfectly reciprocal numerical manner. In the controls, pH increased from 6.07 at day 0 to 6.74 in S and 6.84 in NS at day 28, while titratable acidity decreased to 0.17% and 0.16% lactic acid equivalent, respectively. The 3% celery formulation reached the highest terminal pH (7.25 in S and 7.35 in NS) and the lowest titratable acidity (0.12% and 0.11%). By contrast, R3 and BC3 maintained pH values of 6.18–6.32 and titratable acidity of 0.27–0.29%.

The larger acid reserve of the fruit-powder formulations is consistent with the acidic composition measured for the rosehip and blackcurrant powders and with previous studies reporting acidification by fruit-derived ingredients in meat matrices [16,20,47,48]. The concurrent rise in pH and decline in titratable acidity during storage may reflect consumption or neutralization of organic acids and accumulation of basic nitrogenous compounds. Because individual organic acids and volatile basic nitrogen were not quantified, these mechanisms are presented as plausible explanations rather than directly demonstrated pathways.

### 3.2. Cooking Loss and Proximate Composition

**Figure 2:**
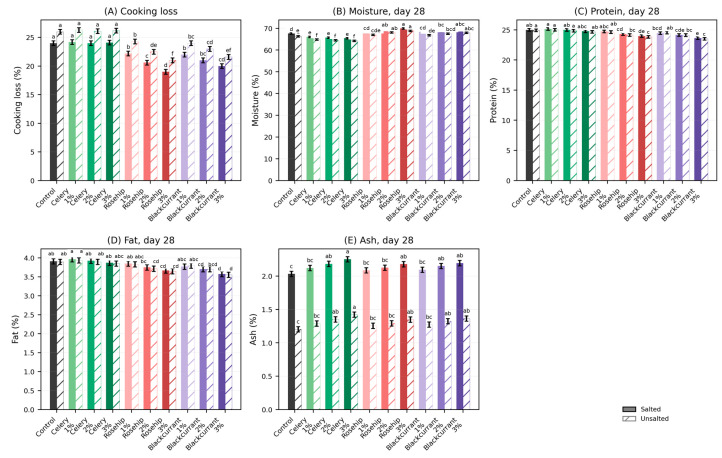
Cooking loss (**A**) and day-28 moisture (**B**), protein (**C**), fat (**D**), and ash (**E**) in salted and unsalted sous-vide pheasant meat. Bars represent means ± SD of three independently packaged samples. Different letters indicate Tukey differences among formulations within the corresponding NaCl series (*p* < 0.05). Open hatched bars represent unsalted samples.

Cooking loss was affected by NaCl condition and formulation (both *p* < 0.001), with no NaCl × formulation interaction (*p* = 0.993). Mean cooking loss was 22.11% in S and 24.10% in NS. R3 showed the lowest values (19.0% in S and 21.0% in NS), followed by BC3 (20.0% and 21.6%), whereas the controls were 24.0% and 26.0%. NaCl can increase ionic strength, promote partial solubilization of myofibrillar proteins, and improve water immobilization during heating [49,50]. Plant-derived solids, including fiber-rich fractions, may also contribute to water binding [16,20]. Conversely, acidification can reduce water-holding capacity when proteins approach their isoelectric region. The observed response therefore represents the combined effects of NaCl, plant solids, pH, and protein–polyphenol interactions rather than a single mechanism, as can be observed in Figure 2.

Moisture declined during storage, while protein and fat percentages increased slightly on a wet-mass basis, a pattern consistent with concentration as water was lost rather than synthesis of protein or lipid. Moisture was influenced by NaCl condition, formulation, day, and formulation × day (all *p* < 0.001). R3 retained the greatest moisture at day 28 (70.00% in S and 68.82% in NS), compared with 67.60% and 66.37% in the controls. Protein and fat were also affected by NaCl condition, formulation, and day. The formulation × day interaction was significant for protein (*p* < 0.001) and fat (*p* = 0.043), whereas the remaining two- and three-factor interactions were not significant. The small storage-related increases should be interpreted in conjunction with moisture loss.

Ash was higher in S than NS because NaCl contributed directly to the mineral residue. At day 28, ash ranged from 2.03% in S-Control to 2.25% in S-C3 and from 1.20% in NS-Control to 1.42% in NS-C3. Formulation and storage day also had significant effects (*p* < 0.001). The plant powders provided additional mineral solids, whereas the slight increase during storage was consistent with water-loss concentration. Selected day-28 values are presented in Table 3, and the complete treatment-level dataset is provided in Supplementary File S1.

### 3.3. Instrumental Texture

**Figure 3:**
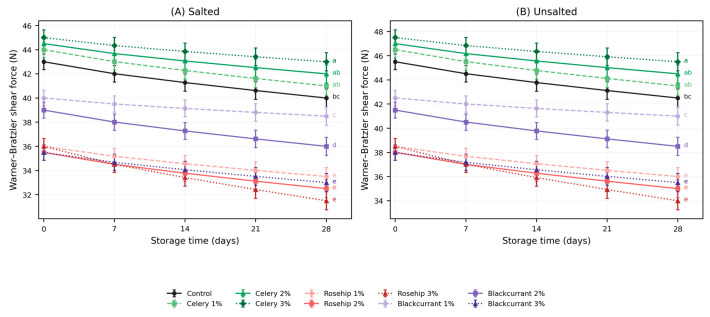
Warner–Bratzler shear force in salted (**A**) and unsalted (**B**) sous-vide pheasant meat during refrigerated storage. Values are means ± SD of three independently packaged samples. Letters adjacent to day-28 points indicate Tukey groupings within the corresponding NaCl series (*p* < 0.05).

Warner–Bratzler shear force was affected by NaCl condition, formulation, and day (all *p* < 0.001); the interactions were not significant, as can be observed in Figure 3. Shear force decreased during storage in all formulations, indicating progressive tenderization. At day 28, the controls reached 40.0 N in S and 42.5 N in NS, whereas R3 reached 31.5 and 34.0 N and BC3 reached 33.0 and 35.5 N. C3 remained the firmest formulation (43.0 and 45.5 N).

Moderate acidification may promote changes in myofibrillar structure and connective-tissue behavior, while phenolic–protein interactions can modify protein aggregation and water distribution [48,49]. The parallel findings of greater retained moisture and lower shear force in R3 and BC3 therefore support a formulation-dependent textural effect, although muscle microstructure and proteolytic activity were not measured directly. Comparable effects of pH, NaCl, plant ingredients, and sous-vide processing on meat texture have been reported previously [37,48,49,50].

### 3.4. Reducing and Radical-Scavenging Capacities, Phenolic-Reactive Compounds, and Lipid Oxidation

**Figure 4:**
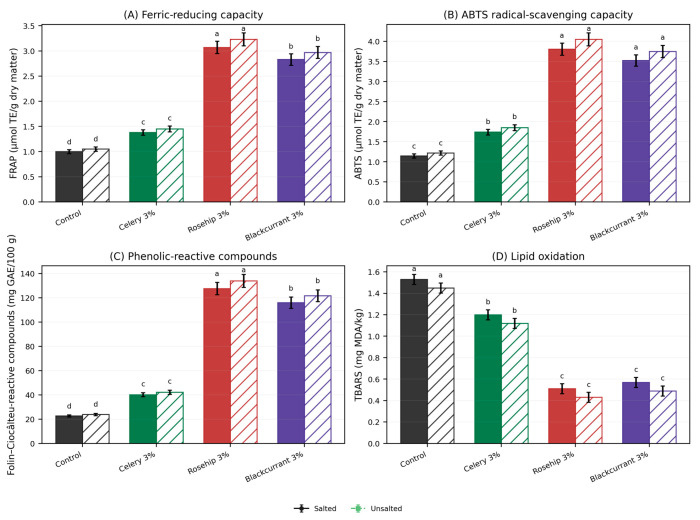
Day-28 ferric-reducing capacity (**A**), ABTS radical-scavenging capacity (**B**), Folin–Ciocâlteu-reactive compounds (**C**), and TBARS (**D**) in selected formulations. Values are means ± SD of three independently packaged samples. Different letters, derived from comparisons among all ten formulations, indicate Tukey differences within each NaCl series and endpoint (*p* < 0.05). Open hatched bars represent unsalted samples.

FRAP, ABTS, and Folin–Ciocâlteu-reactive values declined during storage, but remained substantially higher in the fruit-powder formulations (Figure 4). Formulation and storage day significantly affected all three endpoints (*p* < 0.001). NaCl condition had a smaller but significant effect on all three endpoints. Formulation × day interactions were significant for FRAP, ABTS, and Folin–Ciocâlteu-reactive values (all *p* < 0.001). NaCl × formulation interactions were significant for ABTS (*p* = 0.034) and Folin–Ciocâlteu-reactive values (*p* = 0.014), but not for FRAP (*p* = 0.221); the other NaCl-related interactions were not significant. At day 28, R3 showed FRAP values of 3.07 µmol TE/g dry matter in S and 3.23 µmol TE/g dry matter in NS, compared with 1.00 and 1.05 µmol TE/g dry matter in the controls. The corresponding ABTS values were 3.81 and 4.05 µmol TE/g dry matter for R3 and 1.15 and 1.22 µmol TE/g dry matter for the controls. R3 also retained 127.58–133.95 mg GAE/100 g phenolic-reactive compounds, followed by BC3 at 115.94–121.74 mg GAE/100 g.

ABTS provided a radical-scavenging measurement complementary to the electron-transfer-based FRAP assay. Neither capacity assay alone demonstrates inhibition of oxidation in the meat matrix; TBARS was therefore interpreted separately as an index of secondary lipid oxidation.

TBARS increased during storage and was affected by NaCl condition, formulation, day, NaCl × day, and formulation × day (all *p* ≤ 0.001). At day 28, controls reached 1.53 mg MDA/kg in S and 1.45 mg MDA/kg in NS. R3 remained at 0.51 and 0.43 mg MDA/kg, while BC3 remained at 0.57 and 0.49 mg MDA/kg (Table 4). These results support lower accumulation of TBARS-reactive secondary lipid-oxidation products in the high-dose fruit-powder formulations under the tested conditions. They do not, however, establish inhibition of protein oxidation, because protein carbonyls or other protein-oxidation markers were not measured. The findings are consistent with studies of rosehip, berry pomace, and other fruit-derived ingredients in meat products [16,17,18,19,20,51].

**Table 4:** Day-28 reducing capacity, radical-scavenging, phenolic-reactive, and lipid-oxidation results in selected formulations.

Sample	FRAP (µmol TE/g Dry Matter)	ABTS (µmol TE/g Dry Matter)	Phenolic-Reactive Compounds (mg GAE/100 g)	TBARS (mg MDA/kg)
S-SV1	1.00 ± 0.04 ^f^	1.15 ± 0.05 ^f^	22.69 ± 0.91 ^f^	1.53 ± 0.05 ^a^
S-C3	1.38 ± 0.06 ^e^	1.74 ± 0.07 ^e^	40.19 ± 1.61 ^e^	1.20 ± 0.05 ^c^
S-R3	3.07 ± 0.12 ^a^	3.81 ± 0.15 ^a^	127.58 ± 5.10 ^a^	0.51 ± 0.05 ^g^
S-BC3	2.83 ± 0.11 ^ab^	3.53 ± 0.14 ^ab^	115.94 ± 4.64 ^b^	0.57 ± 0.05 ^fg^
NS-SV1	1.05 ± 0.04 ^f^	1.22 ± 0.05 ^f^	23.82 ± 0.95 ^f^	1.45 ± 0.05 ^a^
NS-C3	1.45 ± 0.06 ^e^	1.85 ± 0.07 ^e^	42.19 ± 1.69 ^e^	1.12 ± 0.05 ^c^
NS-R3	3.23 ± 0.13 ^a^	4.05 ± 0.16 ^a^	133.95 ± 5.36 ^a^	0.43 ± 0.05 ^g^
NS-BC3	2.97 ± 0.12 ^ab^	3.75 ± 0.15 ^ab^	121.74 ± 4.87 ^b^	0.49 ± 0.05 ^fg^

Values are means ± SD (*n* = 3 independent packages). Letters are based on comparisons among all ten formulations within each NaCl series. FRAP and ABTS values are expressed on a dry-matter basis.

### 3.5. Nitrate, Nitrite, Sodium Chloride, and Chemical-Spoilage Indicators

**Figure 5:**
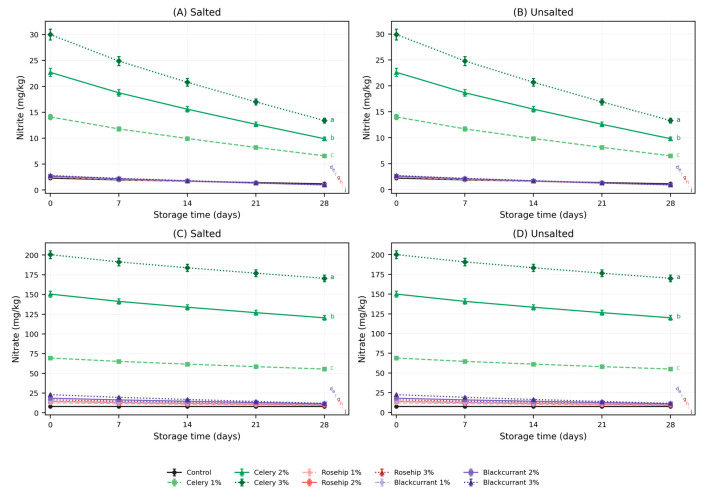
Nitrite (**A**,**B**) and nitrate (**C**,**D**) in salted and unsalted sous-vide pheasant meat during refrigerated storage. Values are means ± SD of three independently packaged samples. Letters adjacent to day-28 values indicate Tukey groupings within the corresponding NaCl series (*p* < 0.05).

Celery produced a clear dose-dependent increase in nitrate and nitrite (Figure 5). Formulation, day, and formulation × day significantly affected both analytes (*p* < 0.001). NaCl condition had a small but significant main effect on nitrite (*p* = 0.014) and nitrate (*p* = 0.030), while NaCl-related interactions were not significant. At day 0, C3 contained approximately 200 mg/kg nitrate and 30 mg/kg nitrite; by day 28, the values were approximately 170–170.5 mg/kg nitrate and 13.3–13.4 mg/kg nitrite. Fruit-powder treatments remained close to the controls. These results identify celery primarily as a nitrate/nitrite-contributing ingredient and are consistent with literature on vegetable-derived curing sources [11,12,52]. Because cured-pigment chemistry was not measured, the results should not be interpreted as proof of equivalent curing-color development.

Analytically recovered NaCl, presented in Table 5, was lower than the nominal 1.8% addition because the measurement was conducted on drained cooked tissue rather than on the complete bag contents. Dissolved salt partitioning into pouch exudate and sample-basis differences therefore provide a plausible explanation for the discrepancy. In S, NaCl increased from 0.83 to 0.95% at day 0 to 0.89–1.06% at day 28; the gradual increase was consistent with concentration during moisture loss. In NS, measured background NaCl remained approximately 0.25–0.27%. NaCl condition, formulation, day, NaCl × formulation, and NaCl × day were significant (*p* < 0.001). The formulation × day interaction was also significant (*p* = 0.008), whereas the three-way interaction was not (*p* = 0.386).

**Table 5:** Analytically recovered sodium chloride in drained cooked tissue at days 0 and 28.

Sample Code	Day 0 (%)	Day 28 (%)
NS-SV1	0.250 ± 0.004	0.261 ± 0.004 ^a^
NS-C1	0.250 ± 0.004	0.261 ± 0.004 ^a^
NS-C2	0.250 ± 0.004	0.267 ± 0.004 ^a^
NS-C3	0.250 ± 0.004	0.264 ± 0.004 ^a^
NS-R1	0.250 ± 0.004	0.262 ± 0.004^a^
NS-R2	0.250 ± 0.004	0.260 ± 0.004 ^a^
NS-R3	0.250 ± 0.004	0.258 ± 0.004 ^a^
NS-BC1	0.250 ± 0.004	0.259 ± 0.004 ^a^
NS-BC2	0.250 ± 0.004	0.258 ± 0.004 ^a^
NS-BC3	0.250 ± 0.004	0.260 ± 0.004 ^a^
S-SV1	0.850 ± 0.012	0.935 ± 0.012 ^c^
S-C1	0.900 ± 0.012	0.987 ± 0.012 ^b^
S-C2	0.920 ± 0.012	1.050 ± 0.012 ^a^
S-C3	0.950 ± 0.012	1.064 ± 0.012 ^a^
S-R1	0.840 ± 0.012	0.933 ± 0.012 ^c^
S-R2	0.840 ± 0.012	0.922 ± 0.012 ^cd^
S-R3	0.830 ± 0.012	0.892 ± 0.012 ^d^
S-BC1	0.840 ± 0.012	0.911 ± 0.012 ^cd^
S-BC2	0.840 ± 0.012	0.901 ± 0.012 ^cd^
S-BC3	0.830 ± 0.012	0.908 ± 0.012 ^cd^

Values are means ± SD (*n* = 3 independent packages). Superscript letters are shown for day-28 values and indicate Tukey differences among formulations within the corresponding NaCl series (*p* < 0.05).

Nessler scores increased during storage. At day 28, both controls and C3 scored 3, whereas R2 and BC2/BC3 scored 1 and R3 remained negative (score 0) in both NaCl series. The ordinal response was directionally consistent with the lower TVC and TBARS values in the higher-dose fruit-powder formulations. Because the test is a semi-quantitative ammonia-related screening procedure, it was not used as a substitute for total volatile basic nitrogen or other quantitative spoilage markers.

### 3.6. Instrumental Color

**Figure 6:**
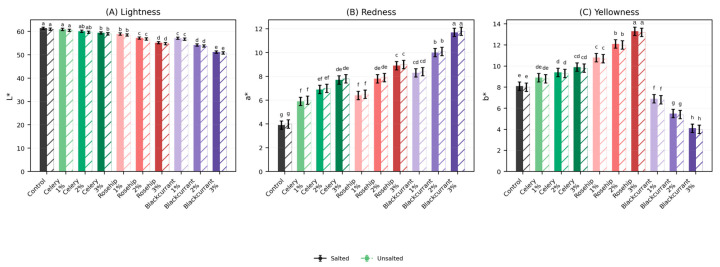
Day-28 CIE L* (**A**), a* (**B**), and b* (**C**) coordinates of salted and unsalted sous-vide pheasant meat. Values are means ± SD of three independently packaged samples. Different letters indicate Tukey differences among formulations within the corresponding NaCl series (*p* < 0.05). Open hatched bars represent unsalted samples.

Instrumental color confirmed substantial formulation effects (Figure 6). L*, a*, and b* were significantly affected by formulation and day (*p* < 0.001); NaCl condition had a smaller significant effect on each coordinate. At day 28, BC3 was the darkest and reddest treatment (L* = 51.2 in S and 50.8 in NS; a* = 11.7 and 11.8), reflecting the strong contribution of blackcurrant pigments. R3 showed the greatest yellowness (b* = 13.3 and 13.2), whereas BC3 showed low b* values (4.1 and 4.0).

These findings demonstrate that colored plant powders materially altered product appearance, a central quality attribute in reformulated meat products, in agreement with prior work on fruit-derived ingredients in meat matrices [15,16,20]. However, instrumental CIELAB coordinates alone do not identify the chemical origin or stability of cured pigments. In particular, the high a* values of blackcurrant formulations may reflect anthocyanin-associated color rather than nitrosomyoglobin formation. The color results should therefore be interpreted as formulation-driven appearance changes, not as validation of nitrite-equivalent curing performance [52].

### 3.7. Microbiological Quality During Refrigerated Storage

**Figure 7:**
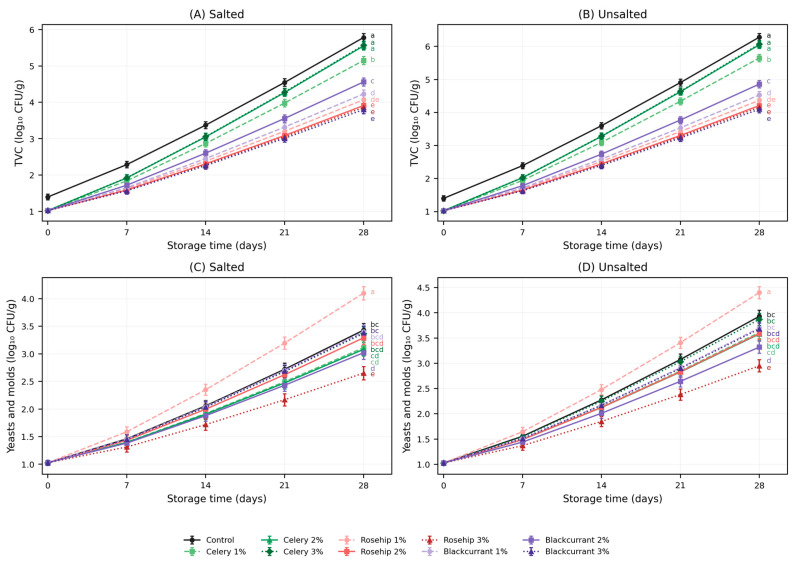
Total viable count (**A**,**B**) and yeast/mold count (**C**,**D**) in salted and unsalted sous-vide pheasant meat during refrigerated storage. Values are means of three independently packaged samples; error bars represent SD. Letters adjacent to day-28 values indicate Tukey groupings within the corresponding NaCl series (*p* < 0.05).

TVC and yeast/mold counts increased during storage (Figure 7). For TVC, NaCl condition, formulation, day, NaCl × formulation, NaCl × day, and formulation × day were significant (*p* ≤ 0.027). For fungi, NaCl condition, formulation, day, NaCl × day, and formulation × day were significant, whereas NaCl × formulation was not (*p* = 0.059). The interaction patterns indicate that treatment differences became progressively more apparent during storage rather than representing a uniform initial reduction.

At day 28, as presented in Table 6, TVC reached 5.78 ± 0.11 log_10_ CFU/g in S-Control and 6.28 ± 0.11 log_10_ CFU/g in NS-Control. R3 and BC3 remained at 3.80–4.16 log_10_ CFU/g, approximately 1.9–2.2 log units below the corresponding controls. The lowest fungal count was observed for R3 (2.65 ± 0.12 in S and 2.95 ± 0.12 log_10_ CFU/g in NS). The lower TVC values in the higher-dose fruit-powder formulations are directionally consistent with reports that acid- and phenolic-rich plant ingredients can delay microbial development in meat systems, although the magnitude depends on ingredient composition and product conditions [15,18,20]. The fungal response was not uniformly favorable at low fruit-powder levels; R1, for example, showed the highest day-28 fungal count. Claims were therefore restricted to the specific higher-dose formulations supported by the data.

Total coliform bacteria and coagulase-positive staphylococci were <2 log_10_ CFU/g, and *C. perfringens* was <1 log_10_ CFU/g, in all day-28 samples. Salmonella spp. and Listeria spp. were not detected in 25 g under the applied screening conditions. These findings describe the tested samples but do not validate safety against germination, growth, or toxin formation by pathogens in extended-shelf-life sous-vide products. A dedicated challenge study would be required for that purpose [8].

**Table 6:** Day-28 microbiological screening results for sous-vide pheasant meat (log_10_ CFU/g).

Sample	TVC	Yeasts/Molds	Coliforms	*C. perfringens*	Coagulase-Positive Staphylococci	*Salmonella* spp.	*Listeria* spp.
NS-SV1	6.28 ± 0.11 ^a^	3.93 ± 0.12 ^b^	<2	<1	<2	ND/25 g	ND/25 g
NS-C1	5.65 ± 0.11 ^b^	3.61 ± 0.12 ^bcd^	<2	<1	<2	ND/25 g	ND/25 g
NS-C2	6.05 ± 0.11 ^a^	3.58 ± 0.12 ^cd^	<2	<1	<2	ND/25 g	ND/25 g
NS-C3	6.07 ± 0.11 ^a^	3.87 ± 0.12 ^bc^	<2	<1	<2	ND/25 g	ND/25 g
NS-R1	4.37 ± 0.11 ^de^	4.40 ± 0.12 ^a^	<2	<1	<2	ND/25 g	ND/25 g
NS-R2	4.21 ± 0.11 ^e^	3.59 ± 0.12 ^bcd^	<2	<1	<2	ND/25 g	ND/25 g
NS-R3	4.16 ± 0.11 ^e^	2.95 ± 0.12 ^e^	<2	<1	<2	ND/25 g	ND/25 g
NS-BC1	4.53 ± 0.11 ^d^	3.70 ± 0.12 ^bc^	<2	<1	<2	ND/25 g	ND/25 g
NS-BC2	4.86 ± 0.11 ^c^	3.32 ± 0.12 ^d^	<2	<1	<2	ND/25 g	ND/25 g
NS-BC3	4.10 ± 0.11 ^e^	3.68 ± 0.12 ^bc^	<2	<1	<2	ND/25 g	ND/25 g
S-SV1	5.78 ± 0.11 ^a^	3.43 ± 0.12 ^b^	<2	<1	<2	ND/25 g	ND/25 g
S-C1	5.15 ± 0.11 ^b^	3.11 ± 0.12 ^bcd^	<2	<1	<2	ND/25 g	ND/25 g
S-C2	5.55 ± 0.11 ^a^	3.08 ± 0.12 ^cd^	<2	<1	<2	ND/25 g	ND/25 g
S-C3	5.57 ± 0.11 ^a^	3.37 ± 0.12 ^bc^	<2	<1	<2	ND/25 g	ND/25 g
S-R1	4.07 ± 0.11 ^de^	4.10 ± 0.12 ^a^	<2	<1	<2	ND/25 g	ND/25 g
S-R2	3.91 ± 0.11 ^e^	3.29 ± 0.12 ^bcd^	<2	<1	<2	ND/25 g	ND/25 g
S-R3	3.86 ± 0.11 ^e^	2.65 ± 0.12 ^e^	<2	<1	<2	ND/25 g	ND/25 g
S-BC1	4.23 ± 0.11 ^d^	3.40 ± 0.12 ^bc^	<2	<1	<2	ND/25 g	ND/25 g
S-BC2	4.56 ± 0.11 ^c^	3.02 ± 0.12 ^d^	<2	<1	<2	ND/25 g	ND/25 g
S-BC3	3.80 ± 0.11 ^e^	3.38 ± 0.12 ^bc^	<2	<1	<2	ND/25 g	ND/25 g

Values are means ± SD (*n* = 3 independent packages). Superscript letters indicate Tukey differences within the corresponding NaCl series. ND/25 g, not detected in 25 g under the analytical conditions used.

### 3.8. Exploratory Laboratory-Panel Evaluation

The day-28 laboratory-panel results showed a consistent descriptive formulation pattern within both sessions (Table 7). In the unsalted session, R3 obtained the highest mean overall sensory-quality score (7.90 ± 0.30), followed by R2 (7.50 ± 0.31) and BC3 (7.40 ± 0.29), whereas the control and C3 scored 5.00 ± 0.61 and 4.20 ± 0.58, respectively. In the salted session, R3 also obtained the highest mean overall sensory-quality score (7.08 ± 0.31), followed by R2 (6.68 ± 0.32) and BC3 (6.56 ± 0.29), whereas the control and C3 scored 4.21 ± 0.59 and 3.44 ± 0.57, respectively. These values are presented as descriptive results for the participating panel rather than as confirmatory treatment comparisons.

The same descriptive ordering was observed for tenderness, juiciness, and flavor. In the unsalted session, R3 averaged 8.10 ± 0.27 for tenderness, 7.30 ± 0.28 for juiciness, and 7.90 ± 0.29 for flavor. In the salted session, the corresponding R3 means were 7.84 ± 0.29, 6.81 ± 0.30, and 6.93 ± 0.28. Celery-containing formulations, particularly C3, showed the lowest mean flavor and overall-quality scores within each session. Although formulation ordering was broadly similar across sessions, the sessions were not used to estimate a sensory NaCl effect or a NaCl × formulation interaction.

The panel pattern was directionally consistent with the instrumental moisture and shear-force findings and with reports that plant ingredients and sous-vide processing can modify meat texture and flavor [37,47,50]. Nevertheless, the assessment involved ten laboratory assessors and one terminal storage point. It therefore characterizes only the tested formulations and panel, does not describe the onset or trajectory of sensory change during storage, and cannot be generalized to population-level consumer preference. Repeated sensory assessment and an independently powered consumer study would be required for those purposes.

### 3.9. Study Limitations

Several limitations define the scope of inference. First, the experiment used one production campaign and three independently packaged sample units per formulation and day; additional raw-material and processing batches are needed to quantify broader biological and manufacturing variability. Second, the commercial powders were characterized for selected compositional attributes, but individual organic acids, phenolic profiles, dietary fiber, and pigment composition were not determined. Third, TBARS assessed secondary lipid-oxidation products, whereas protein oxidation was not measured [51,53]. FRAP, ABTS, and Folin–Ciocâlteu values should therefore remain described as reducing, radical-scavenging, and phenolic-reactive measurements, respectively. Fourth, CIELAB color was determined, but cured-pigment chemistry and color stability after package opening were not assessed. Fifth, the microbiological component was not a pathogen-challenge study and does not validate a 28-day commercial shelf life. Finally, the laboratory-panel assessment involved ten assessors at the terminal storage point only. It did not characterize sensory evolution during storage, was not designed for population-level consumer inference, and, because the salted and unsalted products were assessed in separate sessions, does not support inference regarding a sensory NaCl effect or its interaction with formulation.

## 4. Conclusions

Celery, rosehip, and blackcurrant powders produced distinct technological responses in sous-vide pheasant meat. Celery mainly increased nitrate/nitrite availability and altered color but did not provide the broadest refrigerated-quality profile. Rosehip and blackcurrant, particularly at 2–3%, maintained a larger acid reserve, reduced cooking loss and shear force, increased reducing and radical-scavenging measurements, and were associated with lower TBARS and total viable counts than the controls. R3 generally provided the most favorable combination of moisture retention, low TBARS, low microbial counts, and day-28 laboratory-panel mean scores, while BC3 showed a closely comparable response and the greatest redness.

The findings support further evaluation of fruit-powder formulations for clean-label sous-vide game meat. They do not establish population-level consumer preference, protein-oxidation control, pathogen safety, or a validated commercial shelf life. Confirmation should include multiple independent production batches, pathogen-challenge testing, direct protein-oxidation markers, pigment characterization, repeated sensory time points, and an adequately powered consumer study.

## Figures and Tables

**Table 1:** Measured characteristics of the commercial plant powders used in the formulations.

Powder	Moisture (%)	pH	Titratable Acidity (% Citric Acid E q.)	TPC (mg GAE/100 g)	FRAP (µmol TE/g Dry Matter)	ABTS (µmol TE/g Dry Matter)	Nitrate (mg/kg)	Nitrite (mg/kg)
Celery powder	7.2	5.8	0.25	450	15	18	7800	110
Rosehip powder	6.8	3.4	4.2	9200	135	160	120	<LOQ
Blackcurrant powder	5.9	3.2	3.8	12,800	180	210	90	<LOQ

Values represent batch-specific measurements. “<LOQ” indicates a concentration below the method quantification limit. TPC, total Folin–Ciocâlteu-reactive compounds; TE, Trolox equivalents.

**Table 2:** Experimental formulations applied to sous-vide pheasant meat.

Sample Code	Plant Ingredient	Addition (% *w*/*w*)	Description
NS-SV1	Control	0	Pheasant meat without added NaCl and plant powder
NS-C1	*Apium graveolens*	1	Pheasant meat + 1% celery powder
NS-C2	*Apium graveolens*	2	Pheasant meat + 2% celery powder
NS-C3	*Apium graveolens*	3	Pheasant meat + 3% celery powder
NS-R1	*Rosa canina*	1	Pheasant meat + 1% rosehip powder
NS-R2	*Rosa canina*	2	Pheasant meat + 2% rosehip powder
NS-R3	*Rosa canina*	3	Pheasant meat + 3% rosehip powder
NS-BC1	*Ribes nigrum*	1	Pheasant meat + 1% blackcurrant powder
NS-BC2	*Ribes nigrum*	2	Pheasant meat + 2% blackcurrant powder
NS-BC3	*Ribes nigrum*	3	Pheasant meat + 3% blackcurrant powder
S-SV1	Control	0	Pheasant meat + 1.8% NaCl, without plant powder
S-C1	*Apium graveolens*	1	Pheasant meat + 1.8% NaCl + 1% celery powder
S-C2	*Apium graveolens*	2	Pheasant meat + 1.8% NaCl + 2% celery powder
S-C3	*Apium graveolens*	3	Pheasant meat + 1.8% NaCl + 3% celery powder
S-R1	*Rosa canina*	1	Pheasant meat + 1.8% NaCl + 1% rosehip powder
S-R2	*Rosa canina*	2	Pheasant meat + 1.8% NaCl + 2% rosehip powder
S-R3	*Rosa canina*	3	Pheasant meat + 1.8% NaCl + 3% rosehip powder
S-BC1	*Ribes nigrum*	1	Pheasant meat + 1.8% NaCl + 1% blackcurrant powder
S-BC2	*Ribes nigrum*	2	Pheasant meat + 1.8% NaCl + 2% blackcurrant powder
S-BC3	*Ribes nigrum*	3	Pheasant meat + 1.8% NaCl + 3% blackcurrant powder

**Table 3:** Selected day-28 proximate-composition and shear-force results for the control and 3% plant-powder formulations.

Sample	Moisture (%)	Protein (%)	Fat (%)	Ash (%)	Shear Force (N)
S-SV1	67.60 ± 0.34 ^d^	25.00 ± 0.22 ^ab^	3.91 ± 0.07 ^ab^	2.03 ± 0.04 ^c^	40.00 ± 0.75 ^bc^
S-C3	65.50 ± 0.34 ^e^	24.77 ± 0.22 ^abc^	3.87 ± 0.07 ^abc^	2.25 ± 0.04 ^a^	43.00 ± 0.75 ^a^
S-R3	70.00 ± 0.34 ^a^	23.95 ± 0.22 ^de^	3.67 ± 0.07 ^cd^	2.18 ± 0.04 ^ab^	31.50 ± 0.75 ^e^
S-BC3	69.15 ± 0.34 ^abc^	23.61 ± 0.22 ^e^	3.57 ± 0.07 ^d^	2.19 ± 0.04 ^ab^	33.00 ± 0.75 ^e^
NS-SV1	66.37 ± 0.34 ^e^	24.92 ± 0.22 ^a^	3.89 ± 0.07 ^ab^	1.20 ± 0.04 ^c^	42.50 ± 0.75 ^bc^
NS-C3	64.30 ± 0.34 ^f^	24.67 ± 0.22 ^ab^	3.85 ± 0.07 ^abc^	1.42 ± 0.04 ^a^	45.50 ± 0.75 ^a^
NS-R3	68.82 ± 0.34 ^a^	23.84 ± 0.22 ^c^	3.65 ± 0.07 ^cd^	1.35 ± 0.04 ^ab^	34.00 ± 0.75 ^e^
NS-BC3	67.95 ± 0.34 ^abc^	23.51 ± 0.22 ^c^	3.55 ± 0.07 ^d^	1.36 ± 0.04 ^ab^	35.50 ± 0.75 ^e^

Values are means ± SD (*n* = 3 independent packages). Superscript letters are derived from comparisons among all ten formulations within each NaCl series. Complete treatment-level results for every formulation and storage time are provided in Supplementary File S1.

**Table 7:** Descriptive day-28 quality ratings from the laboratory panel.

Sample	Tenderness	Juiciness	Flavor	Overall Sensory Quality
NS-SV1	6.30 ± 0.59	4.70 ± 0.63	5.10 ± 0.57	5.00 ± 0.61
NS-C1	6.10 ± 0.56	5.20 ± 0.52	4.90 ± 0.58	4.80 ± 0.54
NS-C2	5.90 ± 0.53	4.90 ± 0.47	4.80 ± 0.51	4.60 ± 0.49
NS-C3	5.40 ± 0.62	4.70 ± 0.55	4.70 ± 0.52	4.20 ± 0.58
NS-R1	6.90 ± 0.19	6.60 ± 0.21	7.00 ± 0.18	6.80 ± 0.20
NS-R2	7.50 ± 0.29	6.90 ± 0.32	7.70 ± 0.30	7.50 ± 0.31
NS-R3	8.10 ± 0.27	7.30 ± 0.28	7.90 ± 0.29	7.90 ± 0.30
NS-BC1	6.60 ± 0.26	6.20 ± 0.22	6.80 ± 0.27	6.70 ± 0.24
NS-BC2	7.50 ± 0.41	6.70 ± 0.34	7.60 ± 0.38	7.00 ± 0.33
NS-BC3	8.00 ± 0.31	7.00 ± 0.30	7.50 ± 0.32	7.40 ± 0.29
S-SV1	5.68 ± 0.57	4.19 ± 0.61	4.14 ± 0.55	4.21 ± 0.59
S-C1	5.25 ± 0.53	4.73 ± 0.56	3.91 ± 0.52	3.97 ± 0.54
S-C2	4.92 ± 0.51	4.40 ± 0.49	3.76 ± 0.47	3.80 ± 0.48
S-C3	4.87 ± 0.58	4.15 ± 0.55	3.66 ± 0.50	3.44 ± 0.57
S-R1	6.42 ± 0.22	6.11 ± 0.25	5.97 ± 0.23	5.98 ± 0.24
S-R2	7.27 ± 0.31	6.40 ± 0.33	6.65 ± 0.30	6.68 ± 0.32
S-R3	7.84 ± 0.29	6.81 ± 0.30	6.93 ± 0.28	7.08 ± 0.31
S-BC1	6.33 ± 0.27	5.74 ± 0.24	5.84 ± 0.26	5.86 ± 0.25
S-BC2	6.87 ± 0.34	6.24 ± 0.31	6.57 ± 0.33	6.23 ± 0.32
S-BC3	7.62 ± 0.32	6.47 ± 0.30	6.53 ± 0.31	6.56 ± 0.29

Values are means ± SD from the same ten laboratory assessors within each session. Results are descriptive and apply only to the participating panel and tested samples; no between-session sensory inference was made.

## Data Availability

The original contributions presented in this study are included in the article/Supplementary Material. Further inquiries can be directed to the corresponding author.

## Supplementary Materials

The following supporting information can be downloaded at https://www.mdpi.com/article/10.3390/foods15132369/s1. Supplementary File S1 provides the complete treatment-level aggregate dataset for all formulations and storage times, including means, standard deviations, sample sizes, units, and sample codes; a data dictionary; batch-specific powder-composition results; fixed-effects ANOVA summaries; within-series Tukey groupings for the laboratory endpoints displayed in the manuscript; day-28 enumerated and categorical microbiological screening outcomes; and descriptive day-28 laboratory-panel means and standard deviations. The supplement contains no participant-level sensory scores.
